# Screening for posttraumatic stress disorder in ARDS survivors: validation of the Impact of Event Scale-6 (IES-6)

**DOI:** 10.1186/s13054-019-2553-z

**Published:** 2019-08-07

**Authors:** Megan M. Hosey, Jeannie-Marie S. Leoutsakos, Ximin Li, Victor D. Dinglas, O. Joseph Bienvenu, Ann M. Parker, Ramona O. Hopkins, Dale M. Needham, Karin J. Neufeld

**Affiliations:** 1 Department of Physical Medicine and Rehabilitation, Johns Hopkins School of Medicine, Baltimore, MD USA; 2 Department of Psychiatry and Behavioral Sciences, Johns Hopkins School of Medicine, Baltimore, MD USA; 3 Department of Biostatistics, Johns Hopkins Bloomberg School of Public Health, Baltimore, MD USA; 4 Division of Pulmonary and Critical Care Medicine, Johns Hopkins School of Medicine, Baltimore, MD USA; 5 Department of Psychiatry and Behavioral Sciences, Johns Hopkins School of Medicine, Baltimore, MD USA; 6 Department of Medicine, Pulmonary and Critical Care Division, Intermountain Medical Center, Murray, UT USA; 7 Center for Humanizing Critical Care, Intermountain Health Care, Murray, UT USA; 8 Neuroscience Center and Psychology Department, Psychology Department and Neuroscience Center, Brigham Young University, Provo, UT USA; 9 Outcomes After Critical Illness and Surgery (OACIS) Group, Johns Hopkins School of Medicine, Baltimore, MD USA

**Keywords:** Posttraumatic stress disorder, Critical care, Survivorship, Validation studies, Mental health, Psychometric

## Abstract

**Background:**

Posttraumatic stress disorder (PTSD) symptoms are common in acute respiratory distress syndrome (ARDS) survivors. Brief screening instruments are needed for clinical and research purposes. We evaluated internal consistency, external construct, and criterion validity of the Impact of Event Scale-6 (IES-6; 6 items) compared to the original Impact of Event Scale—Revised (IES-R; 22 items) and to the Clinician Administered PTSD Scale (CAPS) reference standard evaluation in ARDS survivors.

**Methods:**

This study is a secondary analysis from two independent multi-site, prospective studies of ARDS survivors. Measures of internal consistency, and external construct and criterion validity were evaluated.

**Results:**

A total of 1001 ARDS survivors (51% female, 76% white, mean (SD) age 49 (14) years) were evaluated. The IES-6 demonstrated internal consistency over multiple time points up to 5 years after ARDS (Cronbach’s alpha = 0.96; 95% confidence interval (CI) 0.94 to 0.97). The IES-6 demonstrated stronger correlations with related constructs (e.g., anxiety and depression; |r| = 0.32 to 0.52) and weaker correlations with unrelated constructs (e.g., physical function and healthcare utilization measures (|r| = 0.02 to 0.27). Criterion validity evaluation with the CAPS diagnosis of PTSD in a subsample of 60 participants yielded an area under receiver operating characteristic curve (95% CI) of 0.93 (0.86, 1.00), with an IES-6 cutoff score of 1.75 yielding 0.88 sensitivity and 0.85 specificity.

**Conclusions:**

The IES-6 is reliable and valid for screening for PTSD in ARDS survivors and may be useful in clinical and research settings.

## Background

Survivors of acute respiratory distress syndrome (ARDS) and other critical illnesses frequently experience symptoms of posttraumatic stress disorder (PTSD) [1–4]. PTSD symptoms are associated with long-lasting impairments in activities of daily living [5] and quality of life [6, 7]—outcomes of importance to survivors and their families [8, 9]. Thus, detecting clinically meaningful PTSD symptoms is imperative in this patient population. A valid, reliable and brief screening instrument can assist in evaluating PTSD symptoms in ARDS survivors in both clinical and research settings.

The Impact of Event Scale—Revised (IES-R) is a 22-item screening instrument, with demonstrated reliability and validity for measuring PTSD symptoms in ARDS survivors [10]. The prior validation study in ARDS survivors compared the IES-R to the Clinician-Administered PTSD Scale for the DSM-IV (CAPS), a semi-structured clinician interview that serves as a reference standard for PTSD diagnosis. An IES-R score of 1.6 was associated with 100% sensitivity, 85% specificity, and 100% negative and 50% positive predictive values when compared to the CAP-derived clinical diagnosis of PTSD [10].

An abbreviated 6-item version of the IES-R, known as the IES-6, has demonstrated sensitivity (55–96%) and specificity (74 to 99%) for PTSD symptoms in evaluations of survivors of trauma, natural disasters, and personal violence in four Norwegian and Welsh samples [11]. The 73% item reduction from the IES-R to the IES-6 decreases completion time and may increase the ease of use and feasibility of administration in clinical practice and research. However, it is unclear if the IES-6 is reliable and valid for detecting PTSD symptoms in ARDS survivors. Hence, the objective of this analysis is to evaluate the internal consistency, criterion validity, and external construct validity of the IES-6 in ARDS survivors.

## Methods

### Participants

Data used for this analysis were collected as part of two multi-site prospective studies of ARDS survivors: (1) ARDS Network Long-term Outcomes Study (ALTOS) [12, 13] and (2) Improving Care of Acute Lung Injury (ALI) Patients (ICAP) [1]. ALTOS enrolled patients from > 40 hospitals across the USA who participated in four national ARDS Network trials [14–17], with telephone-based follow-up at 6 and 12 months occurring between 2008 and 2014 [12, 13, 18]. The ICAP study evaluated ARDS survivors enrolled from 13 intensive care units (ICUs) at four hospitals in Baltimore, Maryland via in-person follow-up at 3, 6, 12, 24, 36, 48, and 60 months occurring between 2005 and 2012 [1, 19]. The Institutional Review Board of Johns Hopkins University School of Medicine and all participating institutions approved these studies, and patients provided an informed consent*.*

### Measures of PTSD

The IES-R is a 22-item screening instrument assessing PSTD symptoms [20]. Respondents are asked to report how distressed or bothered they are, over the past 7 days, by symptoms related to a specific trauma, using the following scale: “not at all” (item score 0), “a little bit” (score, 1), “moderately” (score, 2), “quite a bit” (score, 3), or “extremely” (score, 4). The IES-6 is an abbreviated 6-item version of the IES-R (Table 1, [11]) with its score calculated as the mean of the six items. The CAPS is a semi-structured interview conducted by a clinician for assessing clinically significant PTSD symptoms and serves as a reference standard for PTSD diagnosis [21].

**Table 1:** IES-6 questions

IES-R* item no.	Item
6	I thought about it when I did not mean to
21	I felt watchful or on-guard
3	Other things kept making me think about it
12	I was aware that I still had a lot of feelings about it, but I didn't deal with them
11	I tried not to think about it
18	I had trouble concentrating

*IES-R: Impact of Events Scale—Revised is a 22 item Post-traumatic Stress Disorder (PTSD) screening questionnaire

### Measures of external construct validity

We hypothesized, a priori, that the IES-6 would correlate with other clinically related constructs (i.e., anxiety symptoms and general mental health measures), as evaluated by the following instruments, administered at the same time as the IES-R in ALTOS and ICAP studies: (1) Short Form Health Survey-36 version 2 (SF-36, [22]) Mental Health Domain and Mental Component Summary scores, (2) Hospital Anxiety and Depression Scale (HADS) Anxiety and Depression Symptom Subscales [23], and (3) EQ-5D-3L [24] Anxiety/Depression Item.

In contrast to the prior related constructs, we hypothesized a priori that IES-6 scores would be more weakly correlated with other clinically unrelated constructs (i.e., physical function and healthcare utilization measures), as evaluated by the following instruments: (1) SF-36 Physical Function (PF) and Role Physical (RF) Domains; (2) EQ-5D-3L Mobility and Self-care items, (3) Functional Performance Inventory—Short Form (FPI-SF) [25] Body Care, Household Maintenance, and Physical Exercise Domains; and (4) healthcare utilization evaluated as ever vs. never using oxygen, x-ray imaging, magnetic resonance imaging, and hemodialysis. All of the aforementioned measures were previously evaluated in ARDS survivors [10, 25–29].

### Measure of criterion validity

Criterion validity of the IES-6 was assessed using the CAPS-based diagnosis of PTSD as previously described.

### Study procedures

In both ALTOS and ICAP studies, trained research staff administered the IES-R at scheduled follow-up assessments [1, 12–14, 19]. Respondents were asked about PTSD symptoms related to their ICU stay for ARDS. The IES-R, SF-36, HADS, EQ-5D-3L, and healthcare utilization survey were asked at follow-up in each of the studies. Only ALTOS participants completed the FPI-SF. As part of a previously reported sub-study [10], 60 of 77 (78%) participants from ALTOS and ICAP completed a telephone-based CAPS diagnostic interview within 1 week of completing the IES-R. Interviewers included a board-certified attending psychiatrist, a fourth-year psychiatry resident, and a medical student who were extensively trained in the administration of the structured interview.

### Statistical analysis

The R statistical software package version 3.5.0 was used for statistical analysis. We calculated the correlation between IES-R and IES-6 scores across time points using a longitudinal random effects model with a random intercept. To allow interpretation of the regression coefficient as a correlation, we standardized both IES-R and IES-6 scores using the mean and standard deviation (SD) of scores at 6 months—the earliest time point shared by both datasets. To evaluate internal consistency as a measure of reliability, Cronbach’s alpha was calculated for IES-R and IES-6 at each follow-up visit. External construct validity was assessed by examining Pearson correlations (calculated via linear mixed effects) between IES-6 and measures of other constructs, expected to be related or unrelated. We used the same method as described above for correlation between IES-6 and IES-R, to calculate correlations over time between IES-6 and measures of these other constructs. We calculated an analogous set of correlations using IES-R to determine if shortening the scale resulted in attenuation of these expected relationships. Finally, criterion validity was assessed by constructing a receiver operating characteristic curve with a diagnosis of PTSD from the CAPS using the pROC R package. The optimal cutoff score for the IES-6 was obtained, and sensitivity, specificity, negative and positive predictive values were calculated for that cutoff score.

## Results

### Demographics and functional outcome measures

A total of 1001 ARDS survivors (ALTOS: *n* = 815; ICAP: *n* = 186) were included in this evaluation, with 51% female, 76% white, and a mean (SD) age of 49 (14) years (Table 2). Mean values for the IES-R and IES-6 instruments are reported at each of the follow-up assessments in Table 3. In addition, mean scores for measures of constructs expected to be either related or unrelated with PTSD were derived from all follow-up visits (Table 3).

**Table 2:** Baseline characteristics of ARDS survivors from two multicenter cohort studies

Characteristic	ALTOS^a^ *n* = 815	ICAP^b^ *n* = 186
Male, *n* (%)	389 (48)	105 (56)
Race, *n* (%)		
White	655 (80)	107 (58)
Black	121 (15)	75 (40)
Other	39 (5)	4 (2)
Age mean (SD)^c^	49.5 (14.7)	49.1 (14.1)
Body mass index mean (SD)	30.8 (8.7)	28.2 (7.2)
APACHE II mean (SD)^d^	26.0 (8.4)	23.8 (8.1)
ICU length of stay median (IQR) ^e^	10 (7, 16)	14 (10, 23)
Hospital length of stay mean (IQR)	17 (12, 26)	26 (16, 36)
Sample size at follow-up assessment^f^ (*n*)		
3 months	n/a	186
6 months	804	186
12 months	797	178
24 months	n/a	163
36 months	n/a	145
48 months	n/a	138
60 months	n/a	137
CAPS^g^	35	25

^a^ALTOS: Acute Respiratory Distress Syndrome (ARDS) Network Long-term Outcomes Study; ^b^ICAP: Improving Care of Acute Lung Injury (ALI) Patients; c- Mean (standard deviation); d-APACHE II—Acute Physiology and Chronic Health Evaluation; ^e^Median (Interquartile Range); ^f^ALTOS evaluated patients at 6- and 12-month time points only; the decrease in sample size over time for both ALTOS and ICAP studies was due to ongoing patient mortality along with loss to follow-up; g-CAPS: Clinician Administered PTSD Scale: 35 ALTOS patients with follow-up at 1 year and 25 ICAP patients followed up at 2, 3, 4, or 5 years

**Table 3:** Outcome measures in ARDS survivors from two multicenter cohort studies

Outcome measure	ALTOS^a^ *n* = 815	ICAP^b^ *n* = 186
IES-R^d^ at follow-up mean (SD)^c^		
3 months	n/a	1.1 (0.9)
6 months	1.0 (0.9)	0.9 (0.8)
12 months	1.0 (0.9)	0.9 (0.9)
24 months	n/a	0.9 (0.9)
36 months	n/a	0.8 (0.8)
48 months	n/a	0.7 (0.9)
60 months	n/a	0.7 (0.9)
Across all time points	1 (0.9)	0.9 (0.9)
IES-6^e^ at follow-up mean (SD)		
3 months	n/a	1.1 (0.9)
6 months	1.1 (1.0)	0.9 (0.9)
12 months	1.0 (1.1)	0.9 (0.9)
24 months	n/a	0.9 (0.9)
36 months	n/a	0.8 (0.9)
48 months	n/a	0.7 (0.9)
60 months	n/a	0.7 (0.9)
Across all time points	1.1 (1.0)	0.8 (0.9)
External construct validity (related) mean (SD)^f^		
SF-36^g^ Mental Health Domain	44.5 (14.3)	47.1 (13.1)
SF-36^g^ Mental Component Summary	45.3 (14.86)	47.2 (13.2)
HADS^h^ Anxiety	7.0 (5.1)	5.9 (4.7)
HADS^h^ Depression	6.0 (4.9)	5.1 (4.3)
EQ-5D-3L Anxiety/Depression Item	1.7 (0.7)	1.6 (0.6)
External construct validity (unrelated) mean (SD)		
SF-36^g^ Role Physical Subscale	38.5 (13.0)	39.6 (12.8)
SF-36^g^ Physical Function Subscale	37.0 (13.4)	38.3 (13.0)
EQ-5D-3L Mobility Item	1.6 (0.5)	0.6 (0.5)
EQ-5D-3L Self-Care Item	1.3 (0.5)	0.2 (0.5)
FPI^i^ Body Care domain	2.6 (0.6)	n/a
FPI^i^ Maintain Household	1.9 (0.9)	n/a
FPI^i^ Physical Exercise	1.6 (0.9)	n/a
Healthcare utilization, % of patients utilizing		
Oxygen therapy	18.4	14.9
X-ray	53.1	52.3
MRI^j^	21.6	28.9
CT^k^	27.7	34.0

^a^*ALTOS* Acute Respiratory Distress Syndrome (ARDS) Network Long-term Outcomes Study (evaluated patients at 6- and 12-month time points); ^b^*ICAP* Improving Care of Acute Lung Injury (ALI) Patients; ^c^Mean (standard deviation); ^d^*IES-R* Impact of Event Scale—Revised. ^e^*IES-6* Impact of Events Scale-6. ^f^Construct validity measures include mean values (SD) from visits at all available follow-up time points. ^g^*SF-36* Short Form Healthy Survey-36 Version 2. ^h^*HADS* Hospital Anxiety and Depression Scale; ^i^*FPI* Functional Performance Inventory collected for ALTOS only; ^j^*MRI* magnetic resonance imaging; ^k^*CT* computed tomography

### IES-R/IES-6 correlations and internal consistency

The correlation (95% CI) of the IES-R and the IES-6 was 0.96 (0.94 to 0.97) overall assessments. Internal consistency for the IES-6 was good to excellent over time (Cronbach’s alpha of 0.86 to 0.91; Table 4).

**Table 4:** Internal consistency: Cronbach’s alpha statistic for the IES-R and IES-6

Follow-up visit	IES-R*	IES-6**
3 months	0.94	0.86
6 months	0.95	0.86
12 months	0.96	0.90
24 months	0.95	0.85
36 months	0.95	0.86
48 months	0.96	0.91
60 months	0.96	0.89

*IES-R: Impact of Events Scale—Revised; **IES-6: Impact of Events Scale-6

### External construct validity

The IES-6 was moderately correlated with related measures of mental health constructs, including SF-36 Mental Health Domain and Mental Component Summary (|r|, 0.42; 95% CI, 0.39 to 0.46; and 0.46; 95% CI, 0.42 to 0.49 respectively), the HADS Anxiety Subscale (|r|, 0.52; 95% CI, 0.49 to 0.55), the HADS Depression Subscale (|r|, 0.40; 95% CI, 0.37 to 0.44) and the EQ-5D-3L Anxiety/Depression Item (|r|, 0.32; 95% CI, 0.28 to 0.35). The IES-R 22-item version demonstrated the same pattern of associations with these measures, in both magnitude and direction (Table 5).

**Table 5:** External construct validity (related and unrelated measures) for IES-R and IES-6

	IES-R	IES-6
	*r* (95% CI)*	*r* (95% CI)*
Related measures		
SF-36 Mental Health Domain	−0.45 (− 0.48, − 0.42)	− 0.42 (− 0.46, − 0.39)
SF-36 Mental Component Summary	− 0.48 (− 0.52, − 0.45)	− 0.46 (− 0.49, − 0.42)
EQ-5D-3L—Anxiety/Depression item	0.33 (0.29, 0.37)	0.32 (0.28, 0.35)
HADS—Anxiety Subscale	0.55 (0.52, 0.58)	0.52 (0.49, 0.55)
HADS—Depression Subscale	0.42 (0.39, 0.46)	0.40 (0.37, 0.44)
Unrelated measures		
SF-36 Role Physical Domain	− 0.30 (− 0.34, − 0.26)	− 0.27 (− 0.32, − 0.24)
SF-36 Physical Function Domain	− 0.23 (− 0.26, − 0.19)	− 0.21 (− 0.24, − 0.18)
EQ-5D-3L Mobility	0.16 (0.12, 0.20)	0.15 (0.11, 0.19)
EQ-5D-3L Self-Care	0.12 (0.09, 0.16)	0.12 (0.09, 0.16)
FPI^c^ Body Care	− 0.26 (− 0.30, − 0.21)	−0.22 (− 0.27, − 0.18)
FPI Maintain Household	− 0.28 (− 0.33, − 0.24)	−0.26 (− 0.30, − 0.21)
FPI Physical Exercise	− 0.28 (− 0.32, − 0.23)	−0.24 (− 0.29, − 0.20)
Healthcare Utilization		
Oxygen Therapy	0.05 (0.00, 0.09)	0.04 (0.00, 0.08)
X-ray	0.07 (0.02, 0.12)	0.05 (0.01, 0.10)
MRI	0.03 (− 0.02, 0.08)	0.02 (− 0.02, 0.07)
CT	0.04 (0.01, 0.08)	0.03 (0.02, 0.07)

*r = correlation coefficient computed with longitudinal random effects model. *95% CI* 95% confidence interval. *SF-36* Short Form Healthy Survey-36 Version 2, *HADS* Hospital Anxiety and Depression Scale, *FPI* Functional Performance Inventory collected during ALTOS only, *MRI* magnetic resonance imaging, *CT* computed tomography

When compared to correlations with related constructs (above), the IES-6 demonstrated weaker correlations with constructs hypothesized to be unrelated to mental health (Table 5). Of these measures, healthcare utilization variables were least related to the IES-6 ranging from the utilization of MRI (|r|, 0.02; 95% CI 0.02 to 0.07) to utilization of X-rays (|r|, 0.05; 95% CI 0.01 to 0.10). The IES-6 also had a weak correlation with the EQ-5D-3L Mobility and Self Care items (|r|, 0.15; 95% CI, 0.11 to 0.19; and |r|, 0.12; 95% CI, 0.09 to 0.16, respectively), the SF-36 Role Physical and Physical Function Domains (|r|, 0.27; 95% CI, 0.24 to 0.32; and |r|, 0.21; 95% CI, 0.18 to 0.24, respectively), and the FPI Body Care, Maintain Household, and Physical Exercise domains (|r|, 0.22; 95% CI, 0.18 to 0.27; |r|, 0.26; 95% CI, 0.21 to 0.30; |r|, 0.24; 95% CI, 0.20 to 0.29, respectively). Again, these patterns of correlations were similar in magnitude and direction for the IES-R 22 item version (Table 5).

### Criterion validity of the IES-6

**Fig. 1:**
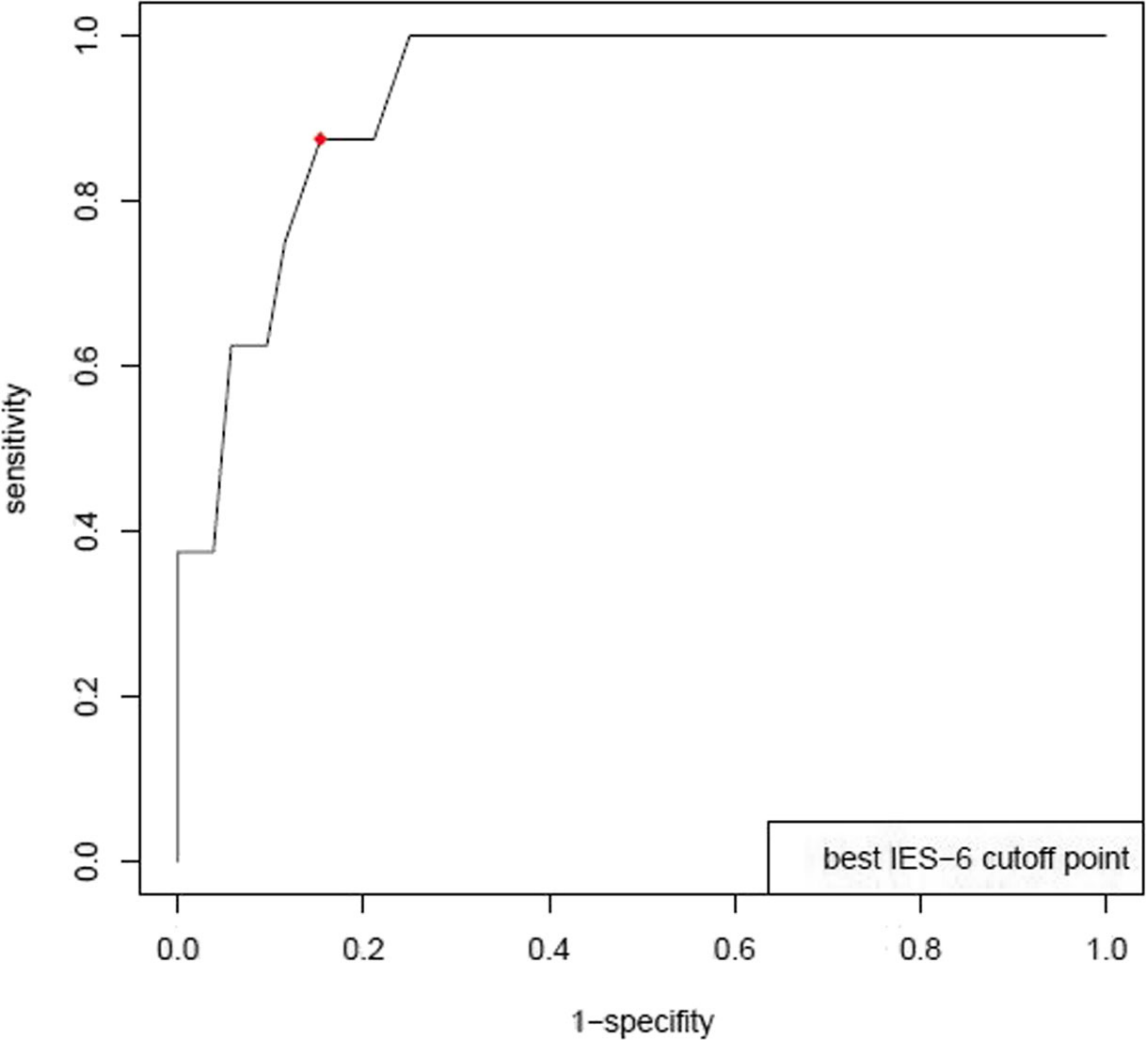
Receiver operating curve for IES-6 versus CAPS for DSM-IV Diagnosis of PTSD (posttraumatic stress disorder); area under the curve (95% confidence interval) = 0.93 (0.86, 1.00); Based on *n* = 60 (13% with PTSD at time of interview, 26% ever experiencing PTSD post-ARDS). Optimal cut-off score for IES-6 is 1.75

Comparison of the IES-6 to the clinician-based current CAP diagnosis of PTSD (13% with PTSD at time of interview and 28% ever experiencing PTSD post-ARDS) yielded an AUROC of 0.93 (95% CI, 0.86 to 1.00) (Fig. 1). The optimal cut-off point is 1.75, resulting in a sensitivity of 0.88, specificity of 0.85 and positive and negative predictive values of 0.47 and 0.98 respectively.

## Discussion

In this evaluation of 1001 ARDS survivors from two multi-site prospective longitudinal studies, the abbreviated IES-6 PTSD screening instrument had good internal consistency with very strong correlations over time with the original 22-item IES-R instrument. External construct validity of the IES-6 was demonstrated by stronger correlations with measures of related constructs (i.e., mental health, anxiety, and depression) over time, and weaker correlations with measures of unrelated constructs (i.e., physical function and healthcare utilization). The IES-6 demonstrated good criterion validity with a cut point of 1.75 resulted in a sensitivity of 0.88 and a specificity of 0.85 when compared to a reference-standard PTSD semi-structured diagnostic clinician interview.

When compared to the PTSD reference standard, the sensitivity and specificity of the IES-6 is slightly lower than the original 22-item IES-R [10], reported as sensitivity = 1.00, specificity = 0.85. However, classification rates remain high with the IES-6, particularly in view of the brevity of the instrument. Use of the IES-6 compared to the IES-R is expected to result in a 75% (4.5 min) reduction in administration time. This important time savings increases the feasibility of PTSD screening for both clinical and research purposes. To our knowledge, the IES-6 is the briefest measure of PTSD symptoms validated in ARDS or ICU survivors, even shorter than the Posttraumatic Stress Syndrome 10-Questions Inventory (PTSS-10), which also has been used in ARDS survivors [30]. More research is needed to compare the reliability and validity of the IES-6 against the PTSS-10.

A scoping review of outcome measurement in studies of critical illness survivors reported substantial heterogeneity, including in the evaluation of PTSD symptoms [31]. Such heterogeneity results in a reduced ability to compare and synthesize existing evidence in order to advance the field [31]. Agreement regarding the measurement of PTSD symptoms is especially important given that an international consensus process identified PTSD as an important outcome domain to be included in all clinical research studies evaluating post-discharge outcomes for acute respiratory failure and ARDS (ARF/ARDS) survivors [32]. This same consensus process specifically recommended the IES-R measure for evaluating PTSD symptoms as part of a minimum core outcomes measurement set (COMS) for ARF/ARDS survivorship research [9]. Our current analysis suggests the abbreviated IES-6, rather than the original IES-R, could improve efficiency, while maintaining adequate measurement properties in screening for PTSD symptoms [9, 32]. Further replication of these findings in other ARDS/ARF populations would be valuable.

Screening for PTSD symptoms may be assisted by knowledge of established risk factors. Studies have consistently demonstrated that light (vs. deep) sedation, severity of illness, and ICU length of stay are not associated with post-discharge symptoms of PTSD in survivors of critical illness [1, 2, 4, 33, 34]. However, common risk factors in this patient population include pre-existing psychiatric comorbidity (e.g., anxiety, depression, and substance misuse), benzodiazepine use in the ICU, and memories of frightening ICU experiences after discharge [1, 2, 4, 33, 35]. Consideration of these issues, along with IES-6 screening, may assist with risk stratification of patients for PTSD symptoms after hospital discharge.

Strengths of this study include its large sample size, with follow up at multiple time points up to 5 years after ARDS onset. Moreover, to our knowledge, this is the first study of the IES-6 in ARDS survivors. However, this evaluation does have some potential limitations. First, the number of participants in the sub-study using the CAPS reference standard was small. However, the confidence interval for the AUC [0.93 (0.86, 1.0)] is relatively precise and suggests this finding is robust. Second, to investigate overlapping variance and test-retest reliability, it is recommended that the original IES-R and abbreviated 6-item version being administered separately at multiple time points [36]. Independent replication is required to address this point. Third, the abbreviated measure reduces the ability to assess PTSD symptom clusters (i.e., avoidance, hyperarousal, intrusion), although the IES-6 does retain 2 items from each cluster [36]. However, the IES-6 can serve as a valid screening tool using a binary cut-off for clinically important PTSD symptoms. Finally, the generalizability of this study is limited given that only ARDS survivors from the United States were evaluated. Further studies of other critical illness survivors in other locations are warranted.

## Conclusion

Based on an evaluation of 1001 ARDS survivors, from two independent multi-site prospective studies longitudinally evaluating patients for up to 5 years, we conclude that the IES-6 is a reliable and valid screening tool for detecting clinically significant symptoms of PTSD. This very brief IES-6 instrument may be of value for PTSD screening during both clinical and research follow-up evaluations.

## Data Availability

Not applicable.

## Abbreviations

ALI: Acute Lung Injury
ALTOS: ARDS Network Long-term Outcomes Study
ARDS: Acute Respiratory Distress Syndrome
CAPS: Clinician-Administered PTSD Scale
FPI-SF: Functional Performance Inventory—Short Form
HADS: Hospital Anxiety and Depression Scale
ICAP: Improving Care of ALI Patients
ICU: Intensive care unit
IES-6: Impact of Event Scale-6
IES-R: Impact of Event Scale—Revised
PTSD: Posttraumatic stress disorder
SF-36: Short Form-36 version 2
