# Correction to: Screening for posttraumatic stress disorder in ARDS survivors: validation of the impact of event Scale-6 (IES-6)

**DOI:** 10.1186/s13054-020-2759-0

**Published:** 2020-02-04

**Authors:** Megan M. Hosey, Jeannie-Marie S. Leoutsakos, Ximin Li, Victor D. Dinglas, O. Joseph Bienvenu, Ann M. Parker, Ramona O. Hopkins, Dale M. Needham, Karin J. Neufeld

**Affiliations:** 1 Department of Physical Medicine and Rehabilitation, Johns Hopkins School of Medicine, Baltimore, MD USA; 2 Department of Psychiatry and Behavioral Sciences, Johns Hopkins School of Medicine, Baltimore, MD USA; 3 Department of Biostatistics, Johns Hopkins Bloomberg School of Public Health, Baltimore, MD USA; 4 Division of Pulmonary and Critical Care Medicine, Johns Hopkins School of Medicine, Baltimore, MD USA; 5 Department of Psychiatry and Behavioral Sciences, Johns Hopkins School of Medicine, Baltimore, MD USA; 6 Department of Medicine, Pulmonary and Critical Care Division, Intermountain Medical Center, Murray, UT USA; 7 Center for Humanizing Critical Care, Intermountain Health Care, Murray, UT USA; 8 Neuroscience Center and Psychology Department, Psychology Department and Neuroscience Center, Brigham Young University, Provo, UT USA; 9 Outcomes After Critical Illness and Surgery (OACIS) Group, Johns Hopkins School of Medicine, Baltimore, MD USA

**Correction to: Crit Care**

**https://doi.org/10.1186/s13054-019-2553-z**

In the publication of this article [1], there is an error in the Abstract. This has now been included in this correction article.

The error in the abstract currently reads:

The IES-6 demonstrated internal consistency over multiple time points up to 5 years after ARDS (Cronbach’s alpha = 0.96; 95% confidence interval (CI) 0.94 to 0.97).

It should instead read in the abstract:

The IES-6 demonstrated internal consistency over multiple time points up to 5 years after ARDS (Cronbach’s alpha = 0.86 to 0.91) and high correlation with the IES-R (0.96; 95% confidence interval (CI): 0.94 to 0.97).
